# Mapping parahippocampal systems for recognition and recency memory in the absence of the rat hippocampus

**DOI:** 10.1111/ejn.12740

**Published:** 2014-09-29

**Authors:** L Kinnavane, E Amin, M Horne, J P Aggleton

**Affiliations:** 1 School of Psychology, Cardiff University70 Park Place, Cardiff, CF10 3AT, UK; 2 Neuroscience & Mental Health Research Institute, Cardiff UniversityCardiff, UK

**Keywords:** area Te2, entorhinal cortex, immediate-early genes, network models, perirhinal cortex

## Abstract

The present study examined immediate-early gene expression in the perirhinal cortex of rats with hippocampal lesions. The goal was to test those models of recognition memory which assume that the perirhinal cortex can function independently of the hippocampus. The c-*fos* gene was targeted, as its expression in the perirhinal cortex is strongly associated with recognition memory. Four groups of rats were examined. Rats with hippocampal lesions and their surgical controls were given either a recognition memory task (novel vs. familiar objects) or a relative recency task (objects with differing degrees of familiarity). Perirhinal Fos expression in the hippocampal-lesioned groups correlated with both recognition and recency performance. The hippocampal lesions, however, had no apparent effect on overall levels of perirhinal or entorhinal cortex c-*fos* expression in response to novel objects, with only restricted effects being seen in the recency condition. Network analyses showed that whereas the patterns of parahippocampal interactions were differentially affected by novel or familiar objects, these correlated networks were not altered by hippocampal lesions. Additional analyses in control rats revealed two modes of correlated medial temporal activation. Novel stimuli recruited the pathway from the lateral entorhinal cortex (cortical layer II or III) to hippocampal field CA3, and thence to CA1. Familiar stimuli recruited the direct pathway from the lateral entorhinal cortex (principally layer III) to CA1. The present findings not only reveal the independence from the hippocampus of some perirhinal systems associated with recognition memory, but also show how novel stimuli engage hippocampal subfields in qualitatively different ways from familiar stimuli.

## Introduction

Medial temporal lobe structures are vital for recognition memory, i.e. the ability to discriminate novel from familiar stimuli. Foremost among these structures is the perirhinal cortex (PRH; Murray, 1996; Brown & Aggleton, 2001; Winters *et al*., 2008). There remains, however, considerable uncertainty about the contributions of the hippocampus (HPC) to recognition memory. Much of this uncertainty arises from lesion studies. Although hippocampal lesions sometimes impair behavioural tests of object recognition, many studies have reported no apparent effect (Clark *et al*., 2000; Mumby, 2001; Winters *et al*., 2008; Broadbent *et al*., 2010; Brown *et al*., 2010; Cohen *et al*., 2013). One possible explanation for the frequent lack of evident hippocampal lesion deficits is found within two-process models of recognition memory, which assume that the PRH is independently responsible for familiarity-based recognition (e.g. Aggleton & Brown, 1999; Norman & O'Reilly, 2003; Diana *et al*., 2007). This particular two-process view contrasts with other models, e.g. where interactions between the PRH and HPC more broadly support recognition (Wixted & Squire, 2011), or hierarchical models that emphasise the perceptual role of the PRH (Cowell *et al*., 2010). The present study directly examined the importance of these interactions by measuring the impact of hippocampal lesions on PRH network activity associated with recognition memory.

Expression of the immediate-early gene (IEG) c-*fos* provides a signal of neuronal activity that is strongly associated with recognition memory. Perirhinal c-*fos* expression increases when animals are passively shown novel stimuli (Zhu *et al*., 1995b, 1996; Wan *et al*., 1999, 2004). In the same studies, hippocampal c-*fos* changes were not observed. Increased perirhinal c-*fos* expression is also seen when rats actively explore and discriminate novel from familiar objects (Albasser *et al*., 2010b), this perirhinal c*-fos* upregulation being required for stable recognition memory (Seoane *et al*., 2012). Active object exploration also reveals networks of c-*fos* expression that link parahippocampal sites with the HPC, patterns that vary depending on whether stimuli are novel or familiar (Albasser *et al*., 2010b). However, the functional significance of these hippocampal activations for recognition memory remains unknown.

To test the involvement of the HPC in modifying PRH activity, rats with excitotoxic lesions of the HPC and control rats which had undergone sham surgery, explored pairs of objects (one novel; one familiar) over multiple recognition trials (novel object condition). Two other groups (hippocampal and sham lesions) only explored objects made familiar by prior exposure over previous sessions, so testing recency memory (familiar object condition). The initial question was whether the hippocampal lesions affected either recognition or recency memory performance. The next question was whether the hippocampal lesions altered c-*fos* expression levels in the PRH (areas 35 and 36) and lateral entorhinal cortex (LEC). Then, by use of the c-*fos* expression data, networks of inter-correlated parahippocampal sites associated with either recognition memory or recency memory were derived with structural equation modelling (SEM). The impact of hippocampal lesions on these networks was then assessed. The final question concerned the potential role of the entorhinal cortex in regulating how hippocampal subfield activity is differentially affected by novel and familiar objects.

## Materials and methods

### Animals

The subjects were 42 male Lister hooded rats (Harlan). They were housed in pairs under diurnal conditions (12 h of light/12 h of dark). Rats were ∼ 12 months old at the beginning of the c-*fos* imaging study. During behavioural testing, they were food-restricted so that they remained close to 85% of their free-feeding body weight. Water was available *ad libitum* throughout. These rats had previously received either hippocampal lesions (*n* = 22) or sham surgery (*n* = 20). They had been trained on a variety of geometric discriminations in a water maze, a spatial alternation task in a T-maze, and a biconditional learning task in boxes (Albasser *et al*., 2013). All experiments were performed in accordance with the UK Animals (Scientific Procedures) Act, 1986 and associated guidelines, and were approved by local ethical committees at Cardiff University.

### Surgery

The rats were ∼ 3 months old at the time of surgery. All rats were anaesthetised with isoflurane gas. They were then placed in a stereotaxic frame with the incisor bar set at − 3.3 mm, and given analgesia in the form of 0.1 mg/kg Metacam (Boehringer Ingelheim Vetmedica, Germany) administered subcutaneously. To expose the skull, a midline sagittal incision was made in the scalp, and the skin was reflected. A craniotomy was made above the injection sites, and the dura was cut to expose the cortex. The hippocampal lesions (*n* = 22) were made with injections of ibotenic acid (Biosearch Technologies, San Rafael, CA, USA) diluted to 63 mm in phosphate-buffered saline (PBS; 0.1 m, pH 7.4). The ibotenic acid was administered via a 2-μm Hamilton syringe connected to a microinjector (Model 5000; Kopf Instruments) set at a rate of 0.1 μL/min, with a subsequent diffusion time of 2 min. The rats received 14 injections into each hemisphere [for coordinates and volumes, see Iordanova *et al*. (2009)]. The surgical control rats (*n* = 20) were treated in the same way until the dura was exposed. While nothing was infused into the brain, the dura was pierced 14 times per hemisphere with a 25-gauge Microlance needle (Becton Dickinson, Drogheda, Ireland).

### Apparatus

**Fig. 1:**
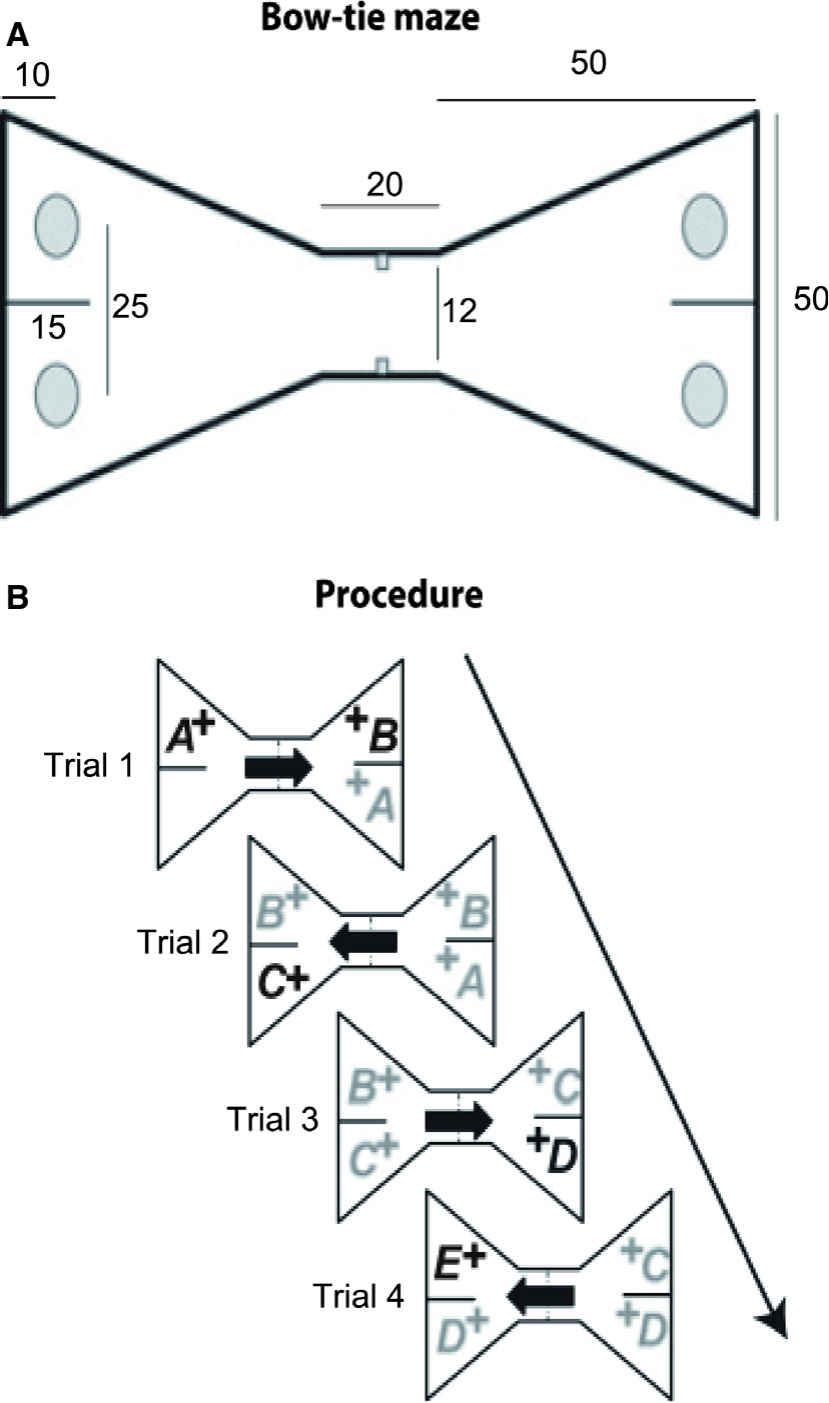
Apparatus (bow-tie maze) used for testing object recognition and object recency memory. (A) Schematic of the test apparatus, with dimensions in centimetres. A sliding door in the centre divides the maze into two halves, so that objects can be placed over the grey food wells in one half while the rat is completing the task in the other half. (B) General procedure showing the order of presentation of objects for recognition memory. All objects are rewarded (+). Thick arrows show the directions of the rats' movements. Group Novel – black type represents novel objects; grey type represents familiar objects. Adapted from Albasser *et al*. (2010b).

Testing took place in a bow-tie maze (Albasser *et al*., 2010a) with steel walls and a wooden floor (Fig. 1A). The maze was 1.2 m in length, 0.5 m in width, and 0.5 m in height. Each end of the maze was triangular in shape, and the apices were joined by a 0.12-m corridor. In the middle of the corridor was an opaque sliding-door that divided the maze in half. Recessed in the floor, by the back wall of each triangular area, were two food wells 3.5 cm in diameter and 2 cm in depth. These food wells were separated by a steel divider that projected 0.15 m into the maze from the centre of the back wall.

### Objects

A total of 147 different junk objects, which varied in colour, shape, size, and texture, were used in the present study. Any object with an obvious scent was excluded. Every object had an identical duplicate. These objects were then equally divided into seven sets of 21 pairs. All objects were large enough to cover a food well, but light enough to be displaced by a rat. All objects were cleaned with alcohol wipes after each session.

### Behavioural testing

#### Animal groups

The rats were divided between two behavioural conditions, creating four groups. The rats that received hippocampal lesions were assigned to either the novel object condition (*n* = 11; HPC Novel) or the familiar object condition (*n* = 11; HPC Familiar). Likewise, the surgical control or ‘sham’ rats were divided between the novel object condition (*n* = 10; Sham Novel) and the familiar object condition (*n* = 10; Sham Familiar).

#### Shared protocol for session 1

The initial session was identical for all four groups. Following successful pre-training in the maze (see Albasser *et al*., 2010a), the rats would run from one end of the maze to retrieve reward pellets placed under objects. A single 45-mg sucrose pellet (Noyes, Lancaster, NH, USA) was placed in each food well, i.e. under every object. Session 1 consisted of 20 trials of 1 min each. At the start of the session, the rat was placed on one side of the maze, which contained a single object (object A). The rat displaced the object to retrieve the single sucrose pellet (Fig. 1B). After 1 min, the experimenter opened the door and the rat ran to the other side of the maze to begin trial 1, in which an identical copy of the now familiar object A was presented along with a novel object (object B). The rat was allowed to freely explore these objects for 1 min. The door was again opened, and the rat would run to the other side of the maze to begin trial 2, in which a copy of the now familiar object (object B) and a novel object (object C) covered the two food wells (Fig. 1B). In trial 3, the familiar object C and the novel object D were used. This running recognition protocol was continued with pairs of objects (one novel; one familiar), covering the baited food wells, until 20 trials were completed. Placement of the novel object on the left or right was counterbalanced.

#### Novel object condition

Both group HPC Novel and group Sham Novel received 13 sessions that were run as described for session 1 (Fig. 1B). Consequently, in each trial, the rats were allowed to explore one novel object and one familiar object (familiar because its copy was seen in the preceding trial) as described above. All objects covered a single sucrose pellet. The first 12 training sessions were given over six consecutive days, i.e. there were two sessions per day. New sets of objects were used for each of the first six sessions, and then used once again in sessions 7–12. The object order and object pairings were not repeated. The final test session was on day 7. The protocol was exactly as described above, except that a novel set of 21 object pairs was used (set 7). As before, each trial comprised one novel object and one object made familiar by its use in the previous trial.

#### Familiar object condition

The test protocol for both familiar object groups (HPC Familiar and Sham Familiar) was the same as described for session 1. In contrast to the novel object condition, the same 21 pairs of objects were then used in all 12 training sessions, although in different orders. This same set of objects (set 7) was then used again for the final test (session 13). Consequently, the objects used in every session for the familiar object condition were the same (set 7) as those used in only the final test session (session 13) of the novel object condition. This comparison task was intended to match the sensorimotor demands of the novel object condition while reducing the impact of object novelty.

#### Analysis of behaviour

The test phase was video-recorded, and behaviours were timed by an experimenter unaware of the surgical history of the individual rats. Object exploration was defined as directing the nose at a distance < 1 cm from the object with the vibrissae moving, and/or touching it with the nose or paws. Behaviour that did not count as exploration included sitting on the object, using the object to rear upwards with the nose pointing at the ceiling, or chewing the object. From these timings, two measures of discrimination were calculated. Index D1 is the amount of time spent exploring the novel object minus the time spent exploring the familiar object (the ‘cumulative D1’ is the sum of the D1 scores for all trials). The second measure, D2, takes into account differences in total exploration times, as D1 is divided by the total amount of exploration given to both objects (Ennaceur & Delacour, 1988). Thus, the D2 ratio can fall between − 1 and + 1. If the ratio is positive, the rat shows a preference for novel objects. The ‘updated D2’ is the D2 score recalculated after each trial.

### Immunohistochemistry

Following completion of the test phase, rats were placed in a dark holding room for 90 min (the rats had been previously been placed in this same dark holding room after each training session). They were then given a lethal overdose of sodium pentobarbital (60 mg/kg, Euthatal; Rhone Merieux), and transcardially perfused with 0.1 m PBS followed by 4% paraformaldehyde in 0.1 m PBS. Brains were removed from the skull, postfixed in 4% paraformaldehyde for 4 h, and then incubated in 25% sucrose at room temperature overnight on a stirrer plate.

The brains were cut in the coronal plane into 40-μm sections with a freezing microtome. A series of one in four sections was collected in PBS, and then stained with cresyl violet. Another series was stained for Fos protein. Tissue from one rat from each of the four groups was processed concurrently to decrease variation. Sections were washed in 0.2% Triton-X 100 in 0.1 m PBS (PBST), once in 0.3% H_2_O_2_ in PBST (to block endogenous peroxidases), then four further times in PBST. Sections were then incubated in primary antibody solution, i.e. rabbit anti-Fos diluted in PBST (1 : 15 000; Calbiochem, EMD Millipore, Cat. no. PC38), for 48 h at 4 °C. Sections were washed four times in PBST, and then incubated in secondary antibody solution, i.e. biotinylated goat anti-rabbit (1 : 200; Vector Laboratories), diluted in 1.5% normal goat serum in PBST for 2 h at room temperature. Sections were washed four times in PBST. They were then incubated in avidin-biotinylated horseradish peroxidase complex in PBST (Elite kit; Vector Laboratories) for 1 h at room temperature. Sections were washed four times in PBST, and then twice in 0.05 m Tris buffer (pH 7.4). All of the above washes were performed for 10 min unless otherwise stated. Finally, diaminobenzidine (DAB Substrate Kit; Vector Laboratories) was used as the chromogen to visualise the location of immunostaining. The reaction was stopped in cold PBS. Sections were mounted onto gelatine-coated slides, dehydrated, and coverslipped.

### Lesion analysis

The extent of the lesion in each hemisphere was drawn onto corresponding coronal plates from a rat brain atlas (Paxinos & Watson, 2005), from bregma − 2.12 mm to − 6.80 mm. These images were then scanned, and the area of damage was calculated with analysis∧d software (Soft-Imaging Systems, Olympus).

### Regions of interest (ROIs)

The ROIs for c-*fos* analysis were the middle and caudal levels of areas 35 and 36 in the PRH (Burwell, 2001), area Te2, and the LEC adjacent to the caudal PRH. Area Te2 was included because it is a key source of visual inputs to the PRH (Burwell & Amaral, 1998; Agster & Burwell, 2009) and because of prior evidence of the importance of this area in the rat brain for visual recognition (Zhu *et al*., 1996; Wan *et al*., 1999; Ho *et al*., 2011). In the sham surgical groups only, additional Fos-positive cell counts were performed in the septal HPC [dentate gyrus (DG), CA1, and CA3]. The septal HPC was chosen because projections from the LEC preferentially terminate in the septal HPC (Ruth *et al*., 1988; Dolorfo & Amaral, 1998), consistent with the finding that subfields in this part of the HPC can be integrated into parahippocampal IEG expression models with good fit (Albasser *et al*., 2010b).

Reflecting the different patterns of inputs from the cortical layers of the LEC to the various hippocampal subfields, separate counts were made in layers II, III and V + VI (combined) of the entorhinal cortex. These distinctions follow the finding that neurons in LEC layer II project to the DG, whereas neurons in LEC layer III project to CA1 (Steward & Scoville, 1976; Insausti *et al*., 1997). There is, however, some inconsistency in the literature regarding the division between layers II and III of the LEC. Some describe layer II as comprising a cell-dense superficial layer IIa and a deeper, slightly less dense layer IIb (Swanson, 1992). Others describe layer IIb as being the superficial component of layer III (Insausti *et al*., 1997; Dolorfo & Amaral, 1998). The latter definition is used in the present study, as this most closely matches the sources of the contrasting inputs to the different hippocampal subfields (Ohara *et al*., 2013).

### Image capture and analysis of c-Fos activation

For each ROI, images were captured from four consecutive sections (each 180 μm apart) from both hemispheres with a × 5 objective lens (numerical aperture of 0.12) on a Leica DMRB microscope and an Olympus DP70 camera. As the field of view was 0.84 × 0.63 mm, cortical regions required one image per section, whereas for the septal HPC multiple images were taken and combined (Microsoft Ice; Microsoft). With analysis∧d software (Soft-Imaging Systems), Fos-positive cells were quantified by counting the nuclei (mean feret of 4–20 μm) stained above a greyscale threshold set ∼ 70 units below the peak grey value for each image measured from a pixel intensity histogram.

### Statistical analysis

Behavioural data ware analysed by use of an anova with two between-subjects factors [surgical group (sham and hippocampal lesion) and behavioural condition (novel object and familiar object)]. Separate analyses examined cumulative D1, updated D2 and total cumulative exploration scores for the final test session, as the measures are not independent. One-tailed, one-sample *t*-tests were applied to the cumulative D1 and updated D2 scores after the final test trial of the test session to determine whether discrimination performance was significantly above chance level (zero) for each group.

To analyse group differences (sham vs. lesion; familiar objects vs. novel objects) in the numbers of Fos-positive cells in the parahippocampal cortices, a two between-subjects factor (lesion type and familiar/novel objects) and one within-subject factor (ROI) anova was performed. This analysis was carried out separately for three regional groupings: (i) divisions within the PRH; (ii) area Te2 and the LEC; and (iii) the various cortical layers of the LEC. The Fos counts in the various hippocampal subfields (sham groups only) were compared by use of a one between-subjects (familiar/novel objects) by one within-subject (ROI) anova.

Pearson product–moment correlation coefficients were calculated for the Fos-positive cell counts in the various parahippocampal sites, as well as with the D2 discrimination ratio. The D2 index was preferred, as it better compensates for individual differences in overall levels of object exploration. The levels of the correlations obtained between the PRH and D2 were also compared between the groups by the use of Fisher's *r*-to-*z* transformation (Zar, 2009).

### SEM

The methods for SEM have been previously described in detail (Poirier *et al*., 2008; Albasser *et al*., 2010b). In brief, structural equation models are multiple-equation regression models that can quantify causal (structural) relationships between sets of variables, including the potential direction of effects. The SEM software package amos 18 (SPSS, Chicago, IL, USA) was used for the path analyses. These analyses estimate parameters on the basis of maximum likelihood estimation (Arbuckle, 2007). The covariance matrices of the regional Fos counts were used to estimate the strength of the relationship (path) between a region and its inputs as set out in the model. The path strength is referred to as the path coefficient (Protzner & McIntosh, 2006). A model is assessed on the basis of how well it replicates the covariance matrices of the observed Fos data. Even when group means for multiple sites remain comparable, the underlying correlations between these same sites may be markedly different (e.g. Poirier *et al*., 2008).

All models tested were based on well-established anatomical connections (Furtak *et al*., 2007; Van Strien *et al*., 2009). The goodness of fit of the data to these anatomical models was then assessed by the use of three indices – chi-square (χ^2^), comparative fit index (CFI), and root mean square error of approximation (RMSEA). The first criterion for a model with good fit was that χ^2^ was non-significant and the ratio of χ^2^ to the degrees of freedom was < 2 (Tabachnick & Fidell, 2001). The CFI and RMSEA were chosen because they have been shown to be most applicable for small sample sizes (Fan & Wang, 1998; Hu & Bentler, 1998). The CFI is based on a baseline comparison with a null model in which no regions are connected (a CFI of > 0.9 is considered to be acceptable). The RMSEA accounts for parsimony in the model, as it estimates the mean lack of fit per degree of freedom (an RMSEA of < 0.08 is considered to be acceptable (Tabachnick & Fidell, 2001)]. Additionally, to ensure that model fit statistics remained robust with small sample sizes, the ratio of regions specified in each model to the number of cases was < 2 for every model tested (Bollen & Long, 1992).

Models were compared by stacking in order to test for group differences in the path coefficients within the same overall model. For stacking, the structural weights of all paths in the model are constrained to be equal across groups, each path is independently unconstrained, and the fit is compared with that of the model in which all paths are constrained (structural weights model). If the model fit when the path is unconstrained is significantly improved, as determined by a χ^2^ difference test, this indicates that the strength of that path differs among the groups (Protzner & McIntosh, 2006).

## Results

### Lesion analysis

Three rats were eliminated from analysis owing to inadequate lesion size; two from group HPC Novel and one from group HPC Familiar. A further rat was removed from group HPC Familiar owing to extensive cortical damage. Thus, the final group numbers were as follows: HPC Familiar, *n* = 9; HPC Novel, *n* = 9; Sham Familiar, *n* = 10; and Sham Novel, *n* = 10.

**Fig. 2:**
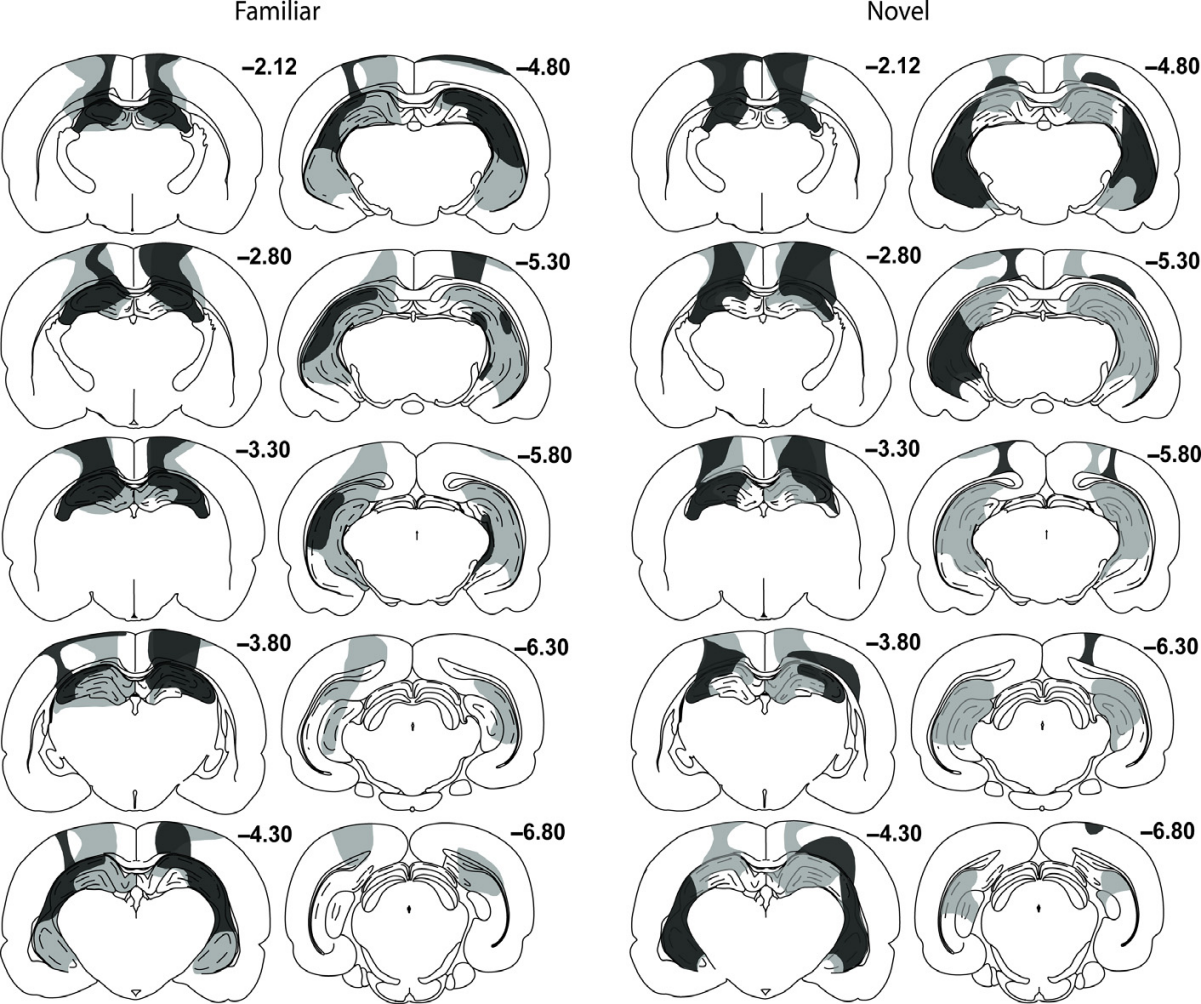
Hippocampal lesion reconstructions – diagrammatic reconstructions of the hippocampal lesions showing the individual cases with the largest (grey) and smallest (black) lesions for group HPC Familiar (left; *n* = 9) and group HPC Novel (right; *n* = 9). The numbers refer to the distance (in millimetres) from bregma. Adapted from Paxinos & Watson (2005).

Figure 2 illustrates the cases with the largest and smallest hippocampal lesions in groups HPC Familiar and HPC Novel. Assessments of total damage to the HPC (excluding the subiculum) ranged from 35 to 79% in group HPC Familiar (median, 61%) and from 29 to 73% in group HPC Novel (median, 50%). It should be noted that these percentages underestimate the amount of actual tissue loss, as they are based on coronal sections and so do not take into account the additional degree of hippocampal shrinkage in the anterior–posterior plane, which was very evident in all cases. The overall percentage of damage to the septal, intermediate and temporal HPC did not distinguish the two groups (*F*_1,16_ = 2.75, *P* = 0.12), although there was proportionately more tissue loss in the septal than in the temporal HPC across both groups (*F*_2,32_ = 8.65, *P* = 0.001). The group by region interaction was close to significant (*F*_2,32_ = 3.21, *P* = 0.054), as there was a tendency for group HPC Familiar to suffer more tissue loss in the intermediate HPC.

The only region to show any consistent sparing in both groups was the most medial portion of the septal DG. In some cases, this sparing extended laterally to encompass the most medial regions of the septal CA1 and CA3. There were also typically small amounts of sparing of the DG, CA1 and CA3 at the temporal pole of the HPC.

In six rats in group HPC Novel, tissue damage extended ventrally to cause very small amounts of thalamic damage. In two cases there was partial bilateral damage to the lateral dorsal thalamic nucleus (LD), in three cases there was unilateral damage to the lateral posterior nucleus, and one case sustained unilateral damage to both the LD and the lateral posterior nucleus, but in contralateral hemispheres. In five rats in group HPC Familiar, there was a very small amount of dorsal thalamic damage; in one there was unilateral LD damage, in three there was bilateral LD damage, and in one there was bilateral damage to the LD accompanied by unilateral anteroventral nucleus damage. All rats showed some cell loss and thinning in cortical regions overlying the HPC (Fig. 2). This cortical involvement varied, but sometimes included the motor cortex, the primary somatosensory area, the parietal region of the posterior association cortex, and the dysgranular retrosplenial cortex.

### Behavioural testing

**Fig. 3:**
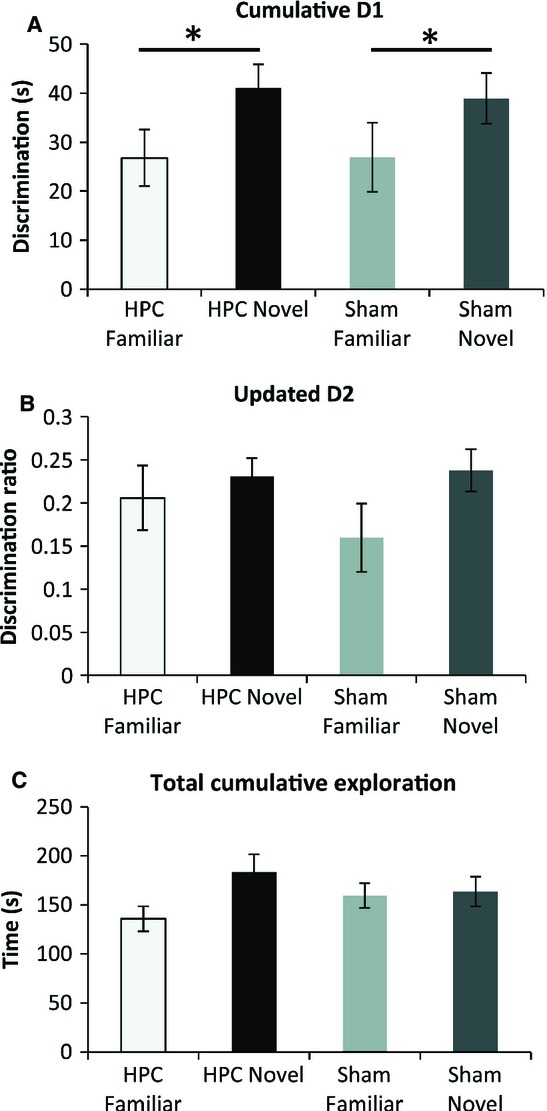
Behavioural measures from the final session (i.e. session 13) of the object recognition test. The graphs depict group performance as measured by – (A) the cumulative D1 recognition index; (B) the updated D2 ratio; and (C) cumulative exploration time for all objects. For D1 and D2, a score of zero indicates a failure to discriminate. All D1 and D2 scores are significantly above zero (one-sample *t*-tests, all *P* < 0.01). *P < 0.05 for novel objects as compared with familiar objects. Data are presented as means ± standard errors of the mean.

Analysis of the cumulative recognition scores from the final session (Fig. 3A) showed the expected higher D1 discrimination indices for the novel object conditions than for the familiar object conditions (*F*_1,34_ = 5.13, *P* = 0.03), irrespective of lesion status. There was no effect of the hippocampal lesions on these discrimination scores across the two conditions (*F* < 1) and no lesion by object type interaction (*F <* 1). All four groups performed above chance on the basis of their cumulative D1 scores (HPC Familiar, *t*_8_ = 4.63, *P* = 0.001; HPC Novel, *t*_8_ = 8.71, *P* ≤ 0.001; Sham Familiar, *t*_9_ = 3.82, *P* = 0.002; Sham Novel, *t*_9_ = 7.47, *P* ≤ 0.001; Fig. 3A), showing that the rats could not only distinguish novel from familiar (novel object condition) objects, but could also distinguish between an object from the previous trial and one from all previous days (familiar object condition).

Analyses based on the D2 index showed a similar pattern (Fig. 3B), except that the discrimination scores failed to differ significantly between the novel object and familiar object conditions (*F*_1,34_ = 3.01, *P* = 0.09). As with D1, there was no evidence of a hippocampal lesion effect (*F* < 1), and nor was there a lesion by condition interaction (*F* < 1). Once again, all four groups performed above chance in this final session (HPC Familiar, *t*_8_ = 5.50, *P* ≤ 0.001; HPC Novel, *t*_8_ = 10.91, *P* ≤ 0.001; Sham Familiar, *t*_9_ = 4.03, *P* = 0.0015; Sham Novel, *t*_9_ = 9.59, *P* ≤ 0.001; Fig. 3B).

Total levels of object exploration in the final session were also calculated (Fig. 3C). This measure was not affected by lesion (*F* < 1) or test condition (*F*_1,34_ = 3.09, *P* = 0.088), or by an interaction between these factors (*F*_1,34_ = 2.18, *P* = 0.15). Finally, correlation coefficients were calculated to determine whether there was an association between lesion size at three levels of the HPC (septal, intermediate, and temporal) and either of the discrimination measures (D1 and D2). No significant correlations were found (all *P* > 0.1), and nor was there any indication that rats with smaller lesions discriminated better in either the novel object or familiar object conditions.

### Fos-positive cell counts

#### Correlations with recognition performance (D2)

The D2 recognition index correlated significantly with the Fos cell counts summed across the PRH (areas 35 and 36 combined) for both group HPC Novel (*r* = − 0.70, *P* = 0.037) and group HPC Familiar (*r* = − 0.81, *P* = 0.008; Table 1). In both cases, the correlation was negative. The corresponding correlations for the remaining groups (Sham Novel, *r* = − 0.46, *P* = 0.18; Sham Familiar, *r* = − 0.30, *P* = 0.39) were not significant. Direct comparisons made between these correlation levels indicated no difference between them, i.e. all *P* > 0.05. When middle and caudal areas 35 and 36 were considered separately, all four subareas had a significant negative correlation with D2 in group HPC Familiar (all *P* < 0.05), as did mid-area 35 in group HPC Novel (*r* = − 0.74, *P* = 0.021). No other ROI in the four groups showed a significant correlation between D2 and Fos-positive cell counts, and nor did the D1 discrimination measure correlate significantly with any ROI.

**Table 1:** Correlations between the Fos-positive cell counts from across all analysed PRH subregions with the discrimination performance (updated D2) for each group

Group	HPC Familiar	HPC Novel	Sham Familiar	Sham Novel
Combined PRH Fos				
*r*-value	− 0.813**	− 0.696*	− 0.304	− 0.457
*P*-value	0.008	0.037	0.393	0.184

The *r*-values are Pearson coefficients. *P < 0.05 and **P < 0.01 for two-tailed correlations.

#### PRH – comparison of Fos counts

**Fig. 4:**
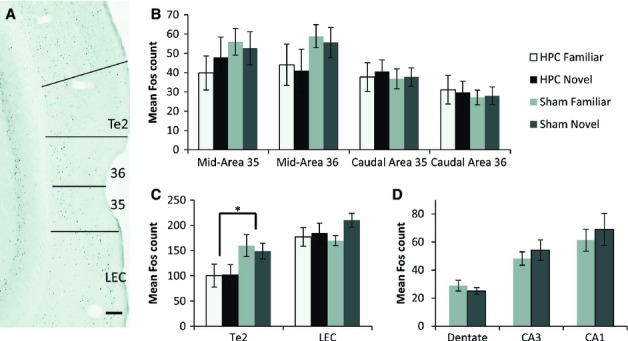
Parahippocampal and hippocampal Fos expression. (A) A representative photomicrograph of a coronal section depicts Fos-positive cells in cortical area Te2, perirhinal areas 35 and 36 and the LEC from a rat in group Sham Novel. Scale bar – 200 μm. (B) Histograms depicting mean counts of perirhinal Fos-positive cells in all four groups in areas 35 and 36 at middle and caudal levels. (C) Histograms depicting mean counts of perirhinal Fos-positive cells in cortical areas adjacent to the PRH– area Te2 and the LEC. (D) Histograms depicting counts of perirhinal Fos-positive cells for the two sham groups in the DG, CA3, and CA1. *P < 0.05 as compared with the appropriate sham condition. Data are presented as means ± standard errors of the mean.

Comparisons across the four perirhinal sites (middle and caudal, areas 35 and 36) showed no overall effect of having a hippocampal lesion (*F* < 1) on Fos-positive cell counts (Fig. 4B). A hippocampal lesion effect was found, however, concerning the interaction with separate counts in the four areas (group by area *F*_3,102_ = 5.29, *P* = 0.002). Simple effects showed that this interaction largely arose from the familiar object condition, as mid-perirhinal areas often contained higher Fos counts in the sham group than the corresponding HPC group, but this difference disappeared in the caudal PRH (Fig. 4B). There was, in addition, an overall effect of ROI (*F*_3,102_ = 27.1, *P* < 0.001), reflecting the consistently lower levels of Fos expression in the more caudal PRH (Fig. 4B). There was no overall effect of behavioural condition (novel vs. familiar objects, *F* < 1); that is, the Fos counts were not higher in the novel object groups. Likewise, there were no significant interactions with the two behavioural conditions.

#### Area Te2 and the LEC – comparison of Fos counts

There was an overall effect of hippocampal surgery (*F*_1,34_ = 4.32, *P* = 0.045), as the rats with lesions had lower Fos counts (Fig. 4C). The surgical group by area interaction (*F*_1,34_ = 4.82, *P* = 0.035) reflected how this reduction in Fos positive cells after hippocampal surgery was essentially confined to area Te2 (Fig. 4C). This Te2 reduction was only significant for the familiar object condition (simple effects *F*_1,34_ = 5.60, *P* = 0.024). There was no overall difference in the Fos counts for the novel object and familiar object groups (*F* < 1), and no interaction with this factor.

Analysis of the cortical layers of the LEC revealed no significant lesion effect (*F* < 1) or effect of behavioural condition (*F*_1,34_ = 2.57, *P* = 0.118) on the numbers of Fos-positive cells in layers II, III, or V + VI. There was also no interaction between these factors (*F*_1,34_ = 1.07, *P* = 0.31).

#### Hippocampal subfields – comparison of Fos counts in sham groups

No significant group differences (novel vs. familiar objects) were found in the Fos counts from the septal hippocampus (DG, CA3, and CA1, *F* < 1; Fig. 4D). There was also no evidence of a group by subfield interaction (*F*_1,21_ = 1.07, *P* = 0.36).

### SEM

The models were derived by using the correlations between the Fos counts found in the different medial temporal sites for the two groups with hippocampal lesions (Table 2) and the two groups that received sham surgery (Table 3). These tables of correlations present probability levels that are not corrected for multiple comparisons, as the individual correlations are of limited significance. More importantly, these same correlations provide the source data for the SEM, in which the fit of the overall model helps to compensate for type 1 errors in the individual correlations that constitute the model. Because of this same concern, it is important that any model must conform to established patterns of connectivity between the ROIs; that is, the number of potential models is constrained.

**Table 2:** Inter-region correlations of Fos-positive cell counts in the two hippocampal lesion groups

HPC Novel									
Brain region	Te2	Mid-area 35	Mid-area 36	Caudal area 35	Caudal area 36	Whole LEC	LEC layer II	LEC layer III	LEC layers V + VI
Te2									
*r*-value	–	**0.636**	**0.607**	0.707*	0.684*	0.698*	**0.019**	0.818**	0.937***
*P*-value	–	**0.066**	**0.083**	**0.033**	**0.042**	**0.036**	**0.960**	**0.007**	**< 0.001**
Mid-area 35									
*r*-value	0.818**	–	0.923***	0.824**	0.669*	**0.659**	**0.214**	**0.638**	0.816**
*P*-value	0.007	–	**< 0.001**	**0.006**	**0.049**	**0.054**	**0.580**	**0.065**	**0.007**
Mid-area 36									
*r*-value	0.661	0.963***	–	0.848**	0.785*	**0.461**	**− 0.012**	**0.482**	0.731*
*P*-value	0.052	< 0.001	–	**0.004**	**0.012**	**0.212**	**0.975**	**0.189**	**0.025**
Caudal area 35									
*r*-value	0.758*	0.943***	0.949***	–	0.930***	**0.627**	**0.191**	**0.639**	0.766*
*P*-value	0.018	< 0.001	< 0.001	–	**< 0.001**	**0.071**	**0.623**	**0.064**	**0.016**
Caudal area 36									
*r*-value	0.774*	0.886**	0.882**	0.972***	–	**0.477**	**0.072**	**0.521**	0.671*
*P*-value	0.014	0.001	0.002	< 0.001	–	**0.194**	**0.853**	**0.150**	**0.048**
Whole LEC									
*r*-value	0.533	0.338	0.264	0.289	0.446	–	–	–	–
*P*-value	0.140	0.373	0.492	0.451	0.229	–	–	–	–
Layer II									
*r*-value	0.023	− 0.326	− 0.394	− 0.336	− 0.129	–	–	**0.550**	**0.233**
*P*-value	0.953	0.391	0.294	0.377	0.740	–	–	**0.125**	**0.546**
Layer III									
*r*-value	0.540	0.279	0.184	0.252	0.421	–	0.709*	–	0.910**
*P*-value	0.134	0.468	0.636	0.514	0.260	–	0.032	–	**0.001**
Layers V + VI									
*r*-value	0.739*	0.788*	0.706*	0.761*	0.786*	–	− 0.010	0.497	–
*P*-value	0.023	0.012	0.033	0.017	0.012	–	0.980	0.173	–
	Te2	Mid-area 35	Mid-area 36	Caudal area 35	Caudal area 36	Whole LEC	LEC layer II	LEC layer III	LEC layers V + VI
HPC Familiar									

The top right diagonal matrix (bold type) relates to data from group HPC Novel, and the bottom left diagonal matrix (normal type) relates to data from group HPC Familiar. The *r*-values are Pearson coefficients. *P < 0.05, **P < 0.01 and ***P < 0.001 for two-tailed correlations (uncorrected for multiple comparisons – see main text). Sites included – area Te2, area 35 and area 36 of the PRH, and the LEC (both as a whole and divided into cortical layers II, III, and V + VI).

**Table 3:** Inter-region correlations of Fos-positive cell counts in the two sham lesion groups

Sham Novel												
Brain region	Te2	Mid-area 35	Mid-area 36	Caudal area 35	Caudal area 36	Whole LEC	LEC layer II	LEC layer III	LEC layers V + VI	DG	CA3	CA1
Te2												
*r*-value	–	**0.617**	0.822**	**0.358**	**0.600**	**− 0.079**	**− 0.529**	**− 0.108**	**0**.662*	**0.121**	**0.639**	**0.598**
*P*-value	–	**0.058**	**0.004**	**0.310**	**0.067**	**0.827**	**0.116**	**0.767**	**0.037**	**0.739**	0.0469*	**0.068**
Mid-area 35												
*r*-value	0.306	–	0.687*	0.756*	**0.392**	**0.476**	**− 0.266**	**0.449**	0.884**	**0.545**	0.748*	**0.619**
*P*-value	0.390	–	**0.028**	**0.011**	**0.263**	**0.165**	**0.457**	**0.193**	**0.001**	**0.104**	**0.013**	**0.056**
Mid-area 36												
*r*-value	0.013	0.77**	–	**0.589**	0.82**	**−** **0.061**	− 0.677*	**− 0.054**	0.757*	**0.338**	0.761*	0.775**
*P*-value	0.972	0.009	–	**0.073**	**0.004**	**0.867**	**0.032**	**0.883**	**0.011**	**0.340**	**0.011**	**0.008**
Caudal area 35												
*r*-value	0.587	0.686*	0.384	–	0.641*	**0.334**	**− 0.416**	**0.360**	0.775**	0.661*	0.801**	0.767**
*P*-value	0.074	0.029	0.274	–	**0.046**	**0.346**	**0.232**	**0.307**	**0.008**	**0.037**	**0.005**	**0.010**
Caudal area 36												
*r*-value	0.618	0.564	0.438	0.917***	–	**− 0.231**	− 0.723*	**− 0.167**	**0.551**	**0.122**	**0.571**	0.669*
*P*-value	0.057	0.090	0.205	< 0.001	–	**0.521**	**0.018**	**0.644**	**0.099**	**0.737**	**0.084**	**0.034**
Whole LEC												
*r*-value	0.270	0.142	− 0.117	− 0.064	− 0.072	–	–	–	–	**0.411**	**0.214**	**0.145**
*P*-value	0.451	0.696	0.747	0.861	0.843	–	–	–	–	**0.238**	**0.553**	**0.689**
Layer II												
*r*-value	− 0.342	− 0.720*	− 0.621	− 0.740*	− 0.742*	–	–	**0.551**	**− 0.591**	**− 0.197**	**− 0.465**	**− 0.486**
*P*-value	0.334	0.019	0.055	0.015	0.014	–	–	**0.099**	**0.072**	**0.585**	**0.175**	**0.155**
Layer III												
*r*-value	0.280	0.216	− 0.154	− 0.057	− 0.111	–	0.222	–	**0.228**	**0.387**	**0.181**	**0.115**
*P*-value	0.433	0.548	0.672	0.875	0.760	–	0.537	–	**0.526**	**0.270**	**0.617**	**0.751**
Layers V + VI												
*r*-value	0.570	0.782**	0.539	0.689*	0.714*	–	− 0.683*	0.443	–	**0.578**	0.782**	0.706*
*P*-value	0.086	0.007	0.108	0.027	0.020	–	0.029	0.200	–	**0.080**	**0.008**	**0.023**
DG												
*r*-value	0.242	0.237	0.401	0.027	0.032	− 0.132	− 0.255	− 0.081	0.083	–	0.789**	0.713*
*P*-value	0.501	0.510	0.251	0.941	0.929	0.716	0.476	0.823	0.820	–	**0.007**	**0.021**
CA3												
*r*-value	0.553	0.509	0.531	0.360	0.371	0.291	− 0.347	0.272	0.536	0.769**	–	0.957***
*P*-value	0.097	0.133	0.115	0.307	0.291	0.414	0.326	0.446	0.110	0.009	–	**< 0.001**
CA1												
*r*-value	0.297	0.468	0.636*	0.298	0.343	0.306	− 0.288	0.178	0.524	0.664*	0.901***	–
*P*-value	0.405	0.173	0.048	0.402	0.332	0.390	0.420	0.623	0.120	0.036	< 0.001	–
	Te2	Mid-area 35	Mid-area 36	Caudal area 35	Caudal area 36	Whole LEC	LEC layer II	LEC layer III	LEC layers V + VI	DG	CA3	CA1
Sham Familiar												

The top right diagonal matrix (bold type) relates to data from group Sham Novel, and the bottom left diagonal matrix (normal type) relates to data from group Sham Familiar. The *r*-values are the Pearson coefficients. *P < 0.05, **P < 0.01 and ***P < 0.001 for two-tailed correlations (uncorrected for multiple comparisons – see main text). Sites included – area Te2, area 35 and area 36 of the PRH, the LEC (both as a whole and divided into cortical layers II, III and V + VI), and hippocampal subfields CA1, CA3, and DG.

#### Parahippocampal models

**Fig. 5:**
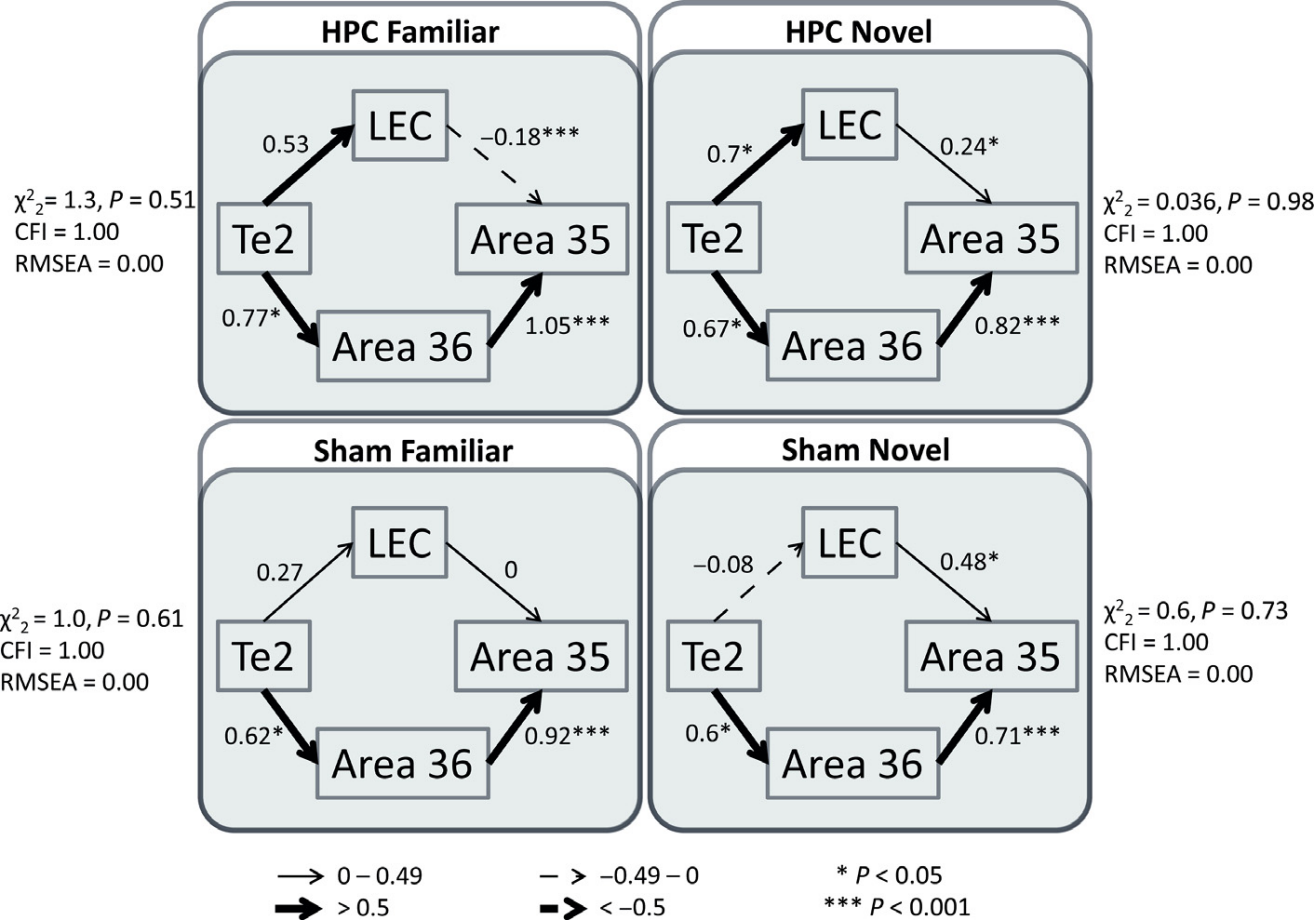
Parahippocampal models for all groups separately. The four models show the optimal parahippocampal interactions derived from SEM for all groups separately – HPC Familiar (top left), HPC Novel (top right), Sham Familiar (bottom left), and Sham Novel (bottom right). The fit is noted beside each model. The strength of the causal influence of each path is denoted both by the thickness of the arrow and by the path coefficient next to that path. Sites depicted – area Te2, area 35 and area 36 of the PRH, and the LEC. *P < 0.05; ***P < 0.001.

Separate models with acceptable fit could be derived from all four groups (Fig. 5). It was striking that the same structural model was optimal for all four groups, whether or not the HPC was intact, and whether the rats explored novel or familiar objects. The only difference concerned the strengths of particular path coefficients. Starting from area Te2, two pathways ran in parallel to area 35 – one pathway via area 36, and the other via the LEC (Fig. 5). In all four groups, the link from area 36 to area 35 had significant path coefficients. When the data from all four groups were combined, the same optimal model emerged but with even higher levels of fit (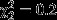, *P* = 0.93, CFI = 1.00, RMSEA = 0.00). This model, for each group individually and with incorporation of all four groups, was also tested with the path directions reversed. This modification generated poorer-fitting models when paths from area 36 to area 35 and the LEC to area 35 were reversed, whereas in the case of the paths between Te2 and area 36, and Te2 and the LEC, path direction did not affect model fit.

Although the data from all four groups fitted the same model, the path strengths between the cortical regions differed (Fig. 5). The four groups were therefore stacked on the same model, in order to compare these differing path strengths. The structural weights of the different paths were constrained such that they had to have the same value in all of the groups; that is, the models were set for each of the groups to be identical. This procedure produced a model of poorer fit than the model in which the structural weights of all paths were free to vary between the groups (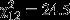, *P* = 0.018), indicating that at least one of the paths differed between the groups. In order to find this path, the structural weights of the different paths were unconstrained individually. The group difference lay in the path from the LEC to area 35, as it was only when this path was unconstrained, in isolation, that there was a significant increase in model fit as compared with the completely constrained model (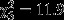, *P* = 0.009).

Examination of Fig. 5 suggests that this significant difference within the same model structure reflects the lower path coefficients for the LEC to area 35 in the two familiar object groups. This difference was tested formally in a series of stacking procedures. These procedures confirmed that novel object vs. familiar object, rather than sham lesion vs. hippocampus lesion, was associated with the change in path coefficients. This analysis initially involved collapsing and stacking the groups across the two between-subjects' factors, i.e. lesion type and object type.

**Fig. 6:**
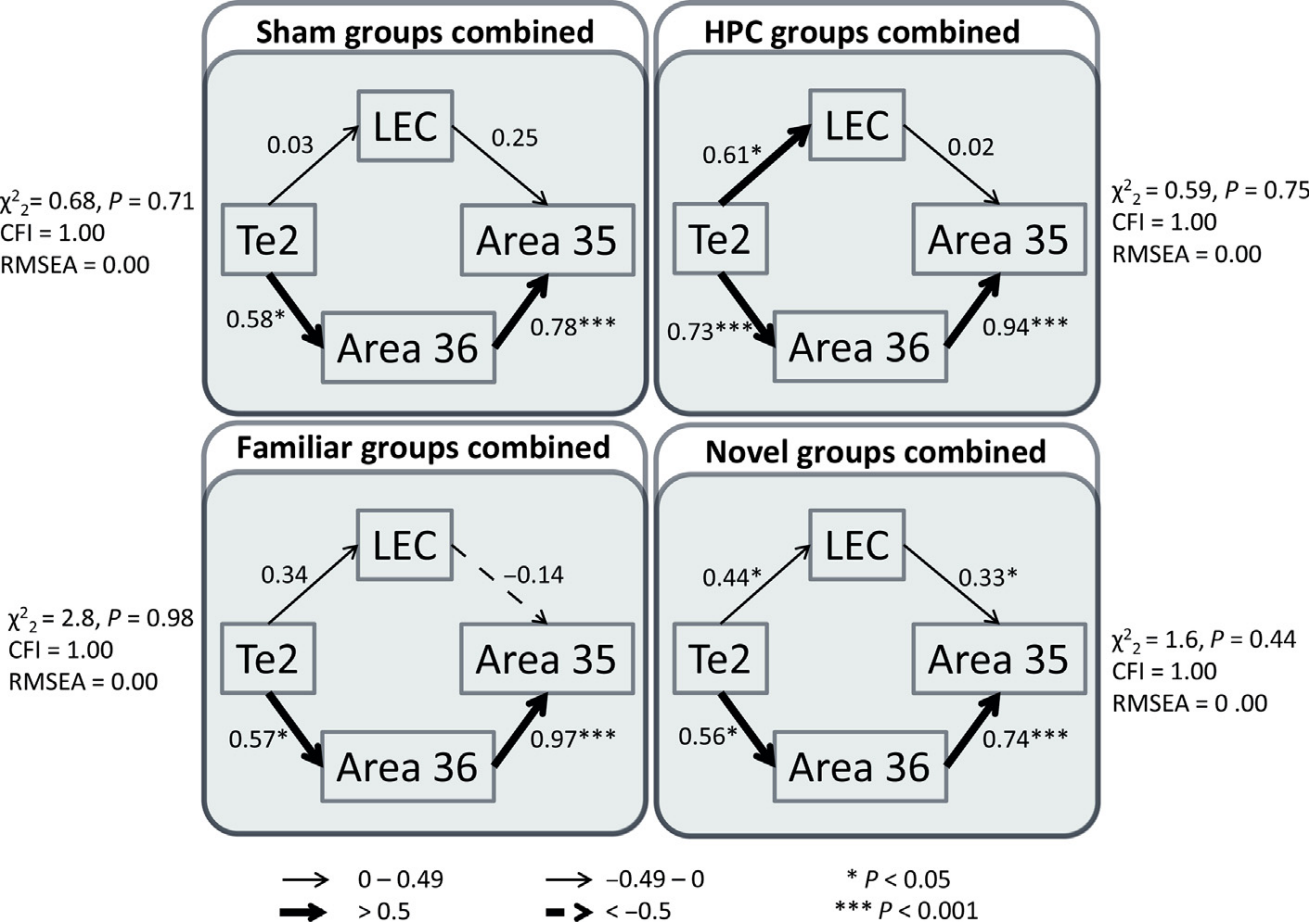
Separate parahippocampal models when the groups are combined according to their surgical status (upper panels) and by their behaviour status (lower panels). The upper panels depict the optimal parahippocampal interactions derived from SEM for both sham surgical groups (upper left) and both HPC lesion groups (upper right), i.e. irrespective of behaviour. The lower panels depict the optimal parahippocampal interactions for groups combined according to their behavioural condition (both familiar object groups, lower left; both novel object groups, lower right), i.e. irrespective of surgery. Model fit is noted beside each model. The strength of the causal influence of each path is denoted both by the thickness of the arrow and by the path coefficient next to that path. Sites depicted – area Te2, area 35 and area 36 of the PRH, and the LEC. *P < 0.05; ***P < 0.001.

Figure 6 (upper) illustrates the separate model fits when the groups were collapsed within each lesion type (sham or hippocampal lesions), i.e. ignoring the behavioural condition. Both the ‘hippocampal’ and ‘sham’ models had good fit, and when the two groups were stacked on the same model in which all path coefficients were free to vary, the fit was not significantly better than the completely constrained model (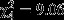, *P* = 0.06), and nor was the model improved by allowing the path from the LEC to area 35 to vary between groups (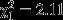, *P* = 0.15). Thus, it can be concluded that the group difference found in the path coefficients for this pathway between the LEC and area 35 was not caused by hippocampal damage. It should be noted that when the path between Te2 and the LEC was unconstrained, the model fit was improved by a small but significant amount (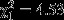, *P* = 0.03).

Finally, the groups were collapsed within the novel object or familiar object conditions (Fig. 6, lower), i.e. ignoring the surgical condition. Once again, both models had good fit. Allowing all the path coefficients to differ between groups significantly improved fit over the completely constrained model (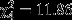, *P* = 0.018), indicating that there is a network difference between the rats exploring novel objects and those exploring familiar ones. Again, paths were unconstrained individually, and this network difference was found in the path coefficients between the LEC and area 35 (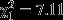, *P* = 0.007), which were positive and significant for the novel object conditions but negative and non-significant for the familiar object conditions (Fig. 6, lower). In order to ensure that this effect was not attributable to an interaction between lesion type and object type, groups HPC Novel and Sham Novel (Fig. 5; top right and bottom right, respectively) were also stacked, but no significant differences were found (data not shown). Thus, it can be concluded that the difference in this path strength reflects the exploration of different object types (novel or familiar objects).

#### Hippocampal–parahippocampal models (sham animals only)

Network models that included the septal hippocampal subfields were calculated for groups Sham Familiar and Sham Novel. The septal HPC was selected because previous research has found that this hippocampal region gives the best-fitting models for c-*fos* expression associated with recognition memory (Albasser *et al*., 2010b). Owing to the addition of more ROIs to the models, areas 35 and 36 were collapsed to a single region (PRH) in order to retain sufficient degrees of freedom for parameters to be estimated.

**Fig. 7:**
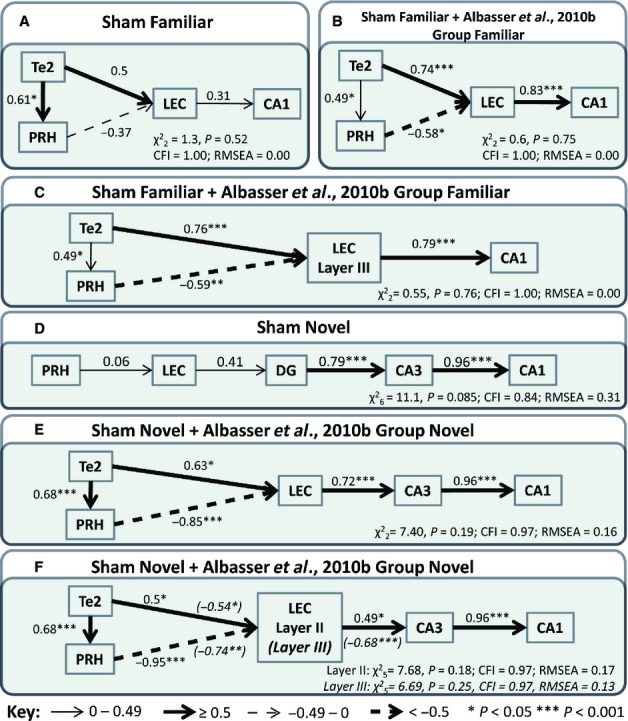
Optimal parahippocampal–hippocampal interactions derived from SEM. (A–C) Models for the familiar object condition. (D–F) Models for the novel object condition. (A) Optimal model for group Sham Familiar obtained from the present data. (B) Optimal model when group Sham Familiar is collapsed with Group Familiar from Albasser *et al*. (2010b). (C) The same model as in B, but the LEC data are now taken only from cortical layer III. (D) Optimal model for group Sham Novel obtained from the present data. (E) Optimal model when group Sham Novel is collapsed with Group Novel from Albasser *et al*. (2010b). (F) The same model as in E, but the LEC data are now taken only from cortical layer II or from layer III (the italicised path coefficients in parentheses relate to layer III). The model fit is provided at the bottom of each panel. The strength of the causal influence of each path is denoted both by the thickness of the arrow and by the path coefficient next to that path. Sites depicted – area Te2, area 35 and area 36 of the PRH, the LEC, and hippocampal subfields CA1, CA3, and DG. *P < 0.05; ***P < 0.001.

The optimal models for groups Sham Familiar and Sham Novel are shown in Fig. 7A and D respectively. Once again, differences between the familiar object and novel object conditions appeared. The optimal model for group Sham Familiar involved a path directly from Te2 to the LEC, and another path from Te2, via the PRH, to the LEC, which, in turn, projects directly to the CA1 subfield. The resulting model had good fit (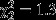, *P* = 0.52, CFI = 1.00, RMSEA = 0.00; Fig. 7A), although only the path from Te2 to the PRH was significant. For group Sham Novel, the best fit was provided by a simplex model from the PRH to the LEC, to the DG, to CA3, and thence to CA1 (Fig. 7D). The fit for this model was, however, relatively poor (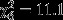, *P* = 0.085, CFI = 0.84, RMSEA = 0.31).

The models for the novel object and familiar object conditions that incorporated the HPC were recalculated with additional correlation data (Fig. 7B and E). This was possible because the two sham groups from the present study constituted a direct replication of a study from the same laboratory using identical protocols and apparatus (Albasser *et al*., 2010b). Although the fine details of the familiar and novel network models derived by Albasser *et al*. (2010b) differ slightly from those in the present study within the parahippocampal cortices (although all involve Te2, the caudal PRH, and the LEC), the pattern of projections to the HPC bear a striking resemblance. The correlational *c-fos* data from Albasser *et al*. (2010b) were accordingly added to the present data to derive models with greater power (Fig. 7B and E).

The optimal familiar object condition model for the combined datasets remained the same as that derived for group Sham Familiar in the present study (Fig. 7B). Not only did this model retain its high fit (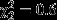, *P* = 0.75, CFI = 1.00, RMSEA = 0.00), but all path coefficients gained significance. The optimal novel object condition model for the combined datasets (Fig. 7E) was, however, different from that described for only group Sham Novel (which did not have high fit; Fig. 7D). Now the fit for the combined model was good (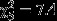, *P* = 0.19, CFI = 0.97, RMSEA = 0.16), and all of the path coefficients gained significance (Fig. 7E). Whereas the parahippocampal components of the combined novel object and combined familiar object groups appeared very similar (Fig. 7B and E), obvious differences occurred in the pathways from the LEC. For novel stimuli, the LEC projects first to CA3 and then to CA1 in the best-fitting model. For familiar stimuli, the pathway from the LEC leads only to CA1 in the best-fitting model.

Layer II of the LEC is known to project to the DG and CA3, whereas layer III projects to CA1 (Steward & Scoville, 1976). Accordingly, the subsequent SEM analyses carried out on these combined models replaced the Fos counts from the whole of the LEC with either layer II or III counts, whereas all other aspects of each model remained the same (Fig. 7C and F). On the basis of the anatomical projections, it might be expected that layer III, but not layer II, of the LEC would provide a model of good fit for the familiar object groups, as it is the principal source of the inputs to CA1. This was found to be the case (Fig. 7C). Replacing all of the LEC with only layer III created a familiar object model of high fit (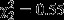, *P* = 0.76, CFI = 1.00, RMSEA = 0.00) in which all path coefficients retained their significance (*P* < 0.05), whereas using only layer II generated a familiar object model of poor fit (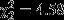, *P* = 0.10, CFI = 0.89, RMSEA = 0.26). For the novel object groups, the converse would be predicted. However, it was found that using only layer II or only layer III generated novel object condition models of acceptable fit (Fig. 7F – layer II, 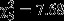, *P* = 0.18, CFI = 0.97, RMSEA = 0.17; layer III, 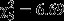, *P* = 0.25, CFI = 0.97, RMSEA = 0.13).

The remaining laminae (V and VI) of the LEC are also of interest, as they are the primary targets for the efferents from the HPC and subiculum (Van Strien *et al*., 2009), as well as the source of projections beyond the temporal lobe to sites such as the prefrontal cortex (Insausti *et al*., 1997). Hence, in the final SEM analyses, Fos counts obtained from combined layers V and VI of the LEC were added to the models in place of counts from the whole LEC (that is, for the novel object condition this analysis was based on the model depicted in Fig. 7E, and for the familiar object condition the analysis was based on the model depicted in Fig. 7B). Interestingly, this generated a model of good fit for the novel object group (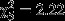, *P* = 0.81, CFI = 1.00, RMSEA = 0.00) but one of poor fit for the familiar object group (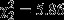, *P* = 0.053, CFI = 0.88, RMSEA = 0.32).

## Discussion

Despite their many interconnections, it has been proposed by some that the PRH can support recognition memory independently of the HPC (e.g. Aggleton & Brown, 1999; Norman & O'Reilly, 2003; Diana *et al*., 2007). To assess this structural independence prediction, the present study examined the impact of hippocampal lesions on PRH activity linked to recognition memory, as measured by c-*fos* expression. To induce c-*fos* expression, rats with either hippocampal lesions or sham surgery actively explored pairs of objects, one novel and one familiar (novel object condition) in the bow-tie maze, a task that is impaired by lesions to the PRH (Albasser *et al*., 2011a,b). The expression of c-*fos* in the PRH, along with area Te2 and various hippocampal subfields, had previously been found to be sensitive to this behavioural manipulation in normal rats (Albasser *et al*., 2010b). Additional information was provided by a familiar object condition, which involved pairs of objects presented at different times in the past, so taxing recency memory (Albasser *et al*., 2010b). Again, there was a group with hippocampal lesions and a group with sham surgery. All four groups in the present study successfully discriminated between the stimuli in their respective novel object and familiar object conditions.

Differences in the overall levels of c-*fos* expression were not observed between the novel object and familiar object conditions [but see Albasser *et al*. (2010b)]. Rather, the two behavioural conditions led to different patterns of inter-correlated c-*fos* expression. At the same time, significant correlations were found between recognition and recency performance and perirhinal Fos counts; these correlations are consistent with c-*fos* activation being closely linked to recognition memory performance (Seoane *et al*., 2012). As these perirhinal correlations were strongest in rats with hippocampal lesions, it is possible that the surgery led to some form of cortical compensation (Cohen *et al*., 2013). Against this view is the finding that these perirhinal correlations did not differ significantly between the surgical and sham groups, and nor did the hippocampal fields show evidence of a significant correlation with object discrimination in the two control groups. Whichever view is correct, the present data still support the notion that the PRH can effectively function independently of the HPC to support recognition memory, although its normal interactions with the HPC are still recognised (Warburton & Brown, 2010).

Irrespective of surgical status, SEM revealed that discriminating novel objects (recognition memory) was associated with particular activity patterns linking area Te2, the PRH, and the LEC. In the novel object condition the surgical control rats had further correlated pathways that linked the LEC to hippocampal area CA3, and thence to CA1 (Fig. 7; these same pathways could not be explored in those rats with hippocampal lesions). Discriminating between familiar objects (recency memory) was also associated with a similar parahippocampal network involving area Te2, the PRH, and the LEC. For the familiar object condition surgical controls, the correlated pathway from the LEC to the HPC went directly to CA1, i.e. not to CA3 as in the novel object condition (Fig. 7).

In the present study, the very short retention delays helped to ensure successful object recognition and object recency discriminations by the surgical control groups. This same feature may also explain the lack of any hippocampal lesion effect on recognition or recency memory performance, although it is only for recency memory that consistent hippocampal lesion deficits are typically reported (Agster *et al*., 2002; Fortin *et al*., 2002; Forwood *et al*., 2005; Hoge & Kesner, 2007; Barker & Warburton, 2011; Albasser *et al*., 2012). It would therefore seem that the parahippocampal cortex can solve simple recency problems, a view supported both by the correlations between perirhinal Fos counts and recency performance, and by the ability of perirhinal units to signal recency differences (Zhu *et al*., 1995a; Xiang & Brown, 1998). The impact of the hippocampal lesions on c-*fos* expression levels was restricted to this same recency memory condition, with decreases in the mid-perirhinal cortex and area Te2. This decrease in perirhinal c-*fos* expression could reflect a disruption of the close cooperation between the PRH and the HPC that is thought to underlie recency memory (Warburton & Brown, 2010; Barker & Warburton, 2011).

The perirhinal Fos counts correlated negatively with the performance index D2 for both recognition and recency memory in the HPC-lesioned groups. The negative sign may seem surprising, given that presenting exclusively novel stimuli increases perirhinal Fos counts (Zhu *et al*., 1995b, 1996; Wan *et al*., 1999, 2004). A likely explanation stems from the fact that recognition memory tests involve the presentation of both novel and familiar stimuli for discrimination. Electrophysiological studies have shown that repeated, i.e. familiar, stimuli are associated with a drop in perirhinal activity, which is thought to provide a familiarity signal (Zhu *et al*., 1995a; Xiang & Brown, 1998; Brown & Aggleton, 2001). The implication is that effective recognition performance relates most to the fall in activity on stimulus repetition, rather than the initial level of activity associated with novel stimuli as such. For this reason, low perirhinal activity may be the hallmark of effective recognition (Montaldi *et al*., 2006). The same logic could also apply to recency discriminations based on relative familiarity (Zhu *et al*., 1995a; Xiang & Brown, 1998).

The initial network analyses, which were largely confined to the parahippocampal region, found that the model with best fit had the same overall structure for all four groups (Fig. 5); that is, it was not affected by hippocampal surgery. Starting from area Te2, two pathways ran in parallel to area 36 and to the LEC, and each then projected to area 35 (Fig. 5). In all four groups, the link from area 36 to area 35 had significant path coefficients, echoing the prevailing connectivity (Burwell & Amaral, 1998). However, stacking the models revealed that the pathway from the LEC to area 35 had stronger effective connectivity in the novel object condition. A combined model based on all four groups was also tested. When the path directions were reversed, poorer-fitting models emerged, except for the paths between Te2 and area 36 and between Te2 and the LEC, where path direction did not affect model fit. This result may reflect the dense reciprocal connections between Te2 and area 36 (Furtak *et al*., 2007), and the moderate reciprocal connections between Te2 and the LEC (Burwell & Amaral, 1998; Agster & Burwell, 2009).

The final network analyses used only the surgical control rats, as the goal was to link parahippocampal with hippocampal activity. The best-fitting models occurred when the present Fos data were combined with those from a previous study (Albasser *et al*., 2010b), which used identical protocols in intact rats. Novel stimuli (recognition) were associated with correlated pathways from the LEC to hippocampal field CA3 (perforant pathway), whereas familiar stimuli (recency) were associated with correlated pathways from the LEC to CA1 (temporo-ammonic pathway). The latter findings extend those studies that have specifically implicated the CA1 subfield in temporal discriminations, and also help to explain the apparent dissociation with CA3 (Gilbert *et al*., 2001; Amin *et al*., 2006; Hoge & Kesner, 2007; Kesner *et al*., 2010). The activation contrast between the perforant and temporo-ammonic pathways has been noted in other IEG studies comparing novel with more familiar stimuli (Poirier *et al*., 2008; Albasser *et al*., 2010b), although these previous studies had less power. In these earlier IEG studies, the best fit for novel stimuli involved the perforant pathway from the entorhinal cortex to the DG, and thence to CA3 (Poirier *et al*., 2008; Albasser *et al*., 2010b). In the present study, the DG was not included in the best novel stimulus model, although there were significant positive correlations between DG and CA3 c-*fos* expression (*P* = 0.007). Additional examination of this novel–familiar pathway dissociation showed that c-fos expression in layer III, but not layer II, of the LEC produced a model of high fit for the familiar object condition. This finding matches the connectivity, as layer III projects to CA1, whereas layer II projects to the DG and CA3 (Steward & Scoville, 1976). More unexpectedly, both layer II and layer III generated models of acceptable fit for the novel object condition.

The different hippocampal subfield interactions for the novel object (CA3) and familiar object (CA1) conditions (Fig. 7) imply that the HPC can help to distinguish novel from familiar stimuli (i.e. support object recognition). There are, however, several caveats. Not only did the hippocampal lesions leave parahippocampal c-*fos* expression levels unaffected for novel stimuli, consistent with spared novelty/familiarity information, but many hippocampal lesion studies have failed to find changes in object recognition memory performance [for reviews, see Mumby (2001), Winters *et al*. (2008), and Brown *et al*. (2010)]. An alternative explanation for this differential hippocampal signalling stems from the fact that when a rat explores an object it does more than register its novelty or familiarity. The rat will spontaneously learn associated information, including its spatial and temporal properties, along with its context (Poucet, 1989; Dix & Aggleton, 1999; Hannesson *et al*., 2004). The extent of this new associative learning should be greatest for novel stimuli (Wagner, 1981). Lesion studies have repeatedly shown that this additional, associative learning requires the HPC (Save *et al*., 1992; Barker & Warburton, 2011), potentially explaining the altered pattern of IEG expression in that structure. This new spatial and temporal information would then be available to support recollection-based recognition (Fortin *et al*., 2004; Diana *et al*., 2007; Easton & Eacott, 2010).

If this analysis is correct, it would be predicted that output routes from the HPC would emerge, reflecting this new associative information. In testing for this possibility, the analysis is constrained by the number of additional ROIs that could be added, owing to the modest sample size, and the number of potential sites, e.g. prefrontal cortex, retrosplenial cortex, and medial diencephalon, which could dilute any such effect (Aggleton, 2012). One output that was considered is that from the HPC to the entorhinal cortex. The major proportion of hippocampal efferents terminate in the deep layers of the LEC (Van Strien *et al*., 2009), so providing the rationale for focusing on only these layers. It was found that, in the novel object condition, Fos counts in combined cortical layers V and VI of the LEC of the sham rats correlated significantly with Fos counts in hippocampal subfields CA1 and CA3, whereas these correlations were not significant in the familiar object condition (Table 3). The resulting SEM provided models with good fit for the novel object conditions but not for the familiar object conditions. Although this preliminary analysis suggests a potential feedback loop in the case of novel stimuli, these hippocampal pathways were seemingly not critical for the c-*fos* responses to novel stimuli in the PRH.

The additional hippocampal learning need not, however, aid familiarity-based recognition memory, as that is context-free (Diana *et al*., 2007). In this account, the PRH is required for object-based information, including familiarity, whereas the HPC supports additional associative learning, in conjunction with the parahippocampal region (Diana *et al*., 2007). This description closely maps onto dual-process models of recognition, which often assume two, largely independent, information streams (Yonelinas, 2002; Norman & O'Reilly, 2003). The present findings also concur with the further assumption that this independence reflects different anatomical substrates (Aggleton & Brown, 1999; Diana *et al*., 2007; Vann *et al*., 2009), with the PRH, in particular, being responsible for familiarity-based recognition, and the HPC being responsible for recollection-based recognition (Brown & Aggleton, 2001; Aggleton *et al*., 2005; Eichenbaum *et al*., 2007; Rudebeck *et al*., 2009).

## Abbreviations

CFI: comparative fit index
DG: dentate gyrus
HPC: hippocampus
IEG: immediate-early gene
LD: lateral dorsal thalamic nucleus
LEC: lateral entorhinal cortex
PBS: phosphate-buffered saline
PBST: 0.2% Triton-X 100 in 0.1 m phosphate-buffered saline
PRH: perirhinal cortex
RMSEA: root mean square error of approximation
ROI: region of interest
SEM: structural equation modelling
